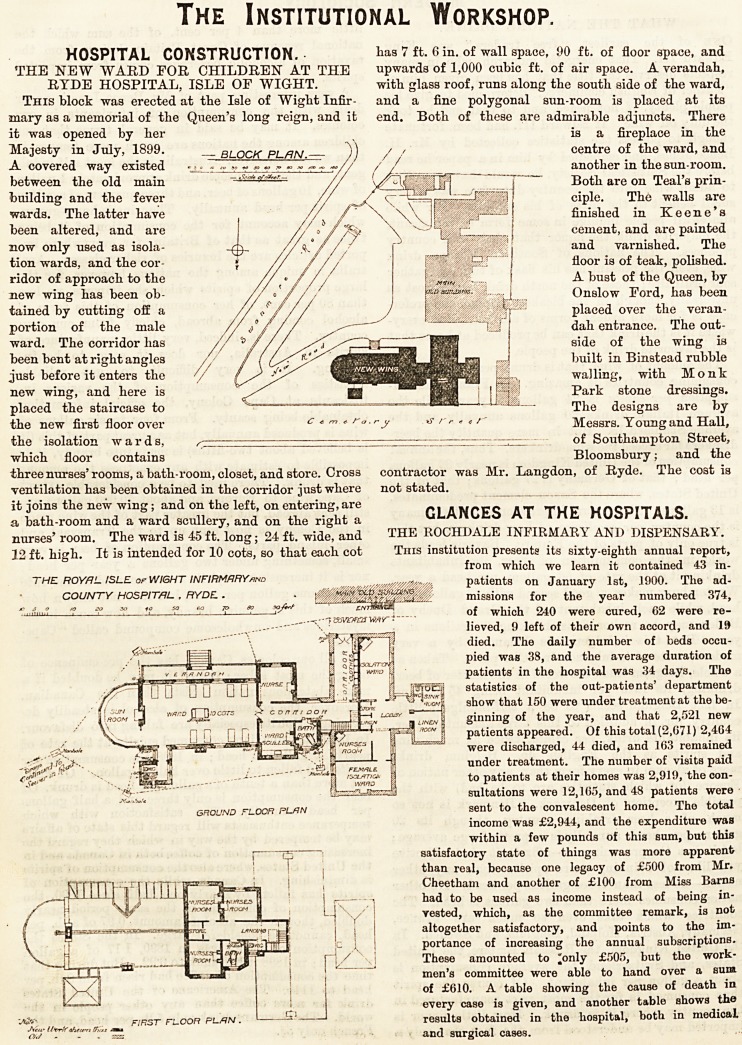# Hospital Construction

**Published:** 1900-09-15

**Authors:** 


					408 THE HOSPITAL. Sept. 15, 1900.
HOSPITAL CONSTRUCTION.
THE NEW WARD FOR CHILDREN AT THE
RYDE HOSPITAL, ISLE OF WIGHT.
This block was erected at the Isle of Wight Infir-
mary as a memorial of tlie Queen's long reign, and it
it was opened by her
Majesty in July, 1899.
A covered way existed
between the old main
building and the fever
wards. The latter have
been altered, and are
now only used as isola-
tion wards, and the cor-
ridor of approach to the
new wing has been ob-
tained by cutting off a
portion of the male
ward. The corridor has
been bent at right angles
just before it enters the
new wing, and here is
placed the staircase to
the new first floor over
the isolation ward s,
which floor contains
three nurses' rooms, a bath-room, closet, and store. Cross
ventilation has been obtained in the corridor just where
it joins the new wing; and on the left, on entering, are
a bath-room and a ward scullery, and on the right a
nurses' room. The ward is 45 ft. long; 24 ft. wide, and
12 ft. high. It is intended for 10 cots, so that each cot
lias 7 ft. 6 in. of wall space, 90 ft. of floor space, and
upwards of 1,000 cubic ft. of air space. A verandah,
with glass roof, runs along tlie south side of the ward,
and a fine polygonal sun-room is placed at its
end. Both of these are admirable adjuncts. There
is a fireplace in the
centre of the ward, and
another in the sun-room.
Both are on Teal's prin-
ciple. The walls are
finished in Keene's
cement, and are painted
and varnished. The
floor is of teak, polished.
A bust of the Queen, by
Onslow Ford, has been
placed over the veran-
dah entrance. The out-
side of the wing is
built in Binstead rubble
walling, with Monk
Park stone dressings.
The designs are by
Messrs. Young and Hall,
of Southampton Street,
Bloomsbury; and the
contractor was Mr. Langdon, of Hyde. The cost is
not stated.
The Institutional Workshop.
HOSPITAL CONSTRUCTION. lias 7 ft. 6 in. of wall space, 90 ffc. of floor space, and
THE NEW "WARD FOR CHILDREN" AT THE upwards of 1,000 cubic ft. of air space. A verandah,
RYDE HOSPITAL, ISLE OF WIGHT. with glass roof, runs along the south side of the ward,
This block was erected at the Isle of Wight Infir- and a fine polygonal sun-room is placed at its
mary as a memorial of the Queen's long reign, and it end. Both of these are admirable adjuncts. There
it was opened by her is a fireplace in the
Majesty in July, 1899.  BLOCK PLAN  )) / ?' centre of the ward, and
A covered way existed ??. ? y ~ *. ?, r ?, r ~,/y / /  another in the sun-room.
between the old main -&<*#?***- // ~ J Both are on Teal's prin-
building and the fever // ciple. The walls are
wards. The latter have //^ \\^ finished in Keene's
been altered, and are //?/n \ cement, and are painted
now only used as isola- //^? // ? f J and varnished. The
tion wards, and the cor- ? // /// Wm c-Xp.: floor is of teak, polished.
ridor of approach to the // <? " //' A bust of the Queen, by
new wing has been ob- // ? / / / Onslow Ford, has been
tained by cutting off a // //// J W$\' placed over the veran-
portion of the male // / / / / - -. . j <]ah entrance. The out-
ward. The corridor has // ^ J // ^ IF1 side of the wing is
been bent at right angles / / / Pf||! jrej built in Binstead rubble
just before it enters the y [J* '&-?>, walling, with Monk
new wing, and here is ' I Park stone dressings.
placed the staircase to   The designs are by
the new first floor over c*m<.ra.ry r r * * /- Messrs. Young and Hall,
the isolation wards, ^ ? ? . ? ? - - - of Southampton Street,
which floor contains Bloomsbury; and the
three nurses'rooms, a bath-room, closet, and store. Cross contractor was Mr. Langdon, of Ryde. The cost is
ventilation has been obtained in the corridor just where not stated.
it joins the new wing; and on the left, on entering, are r Q _ uncmTA. c
a bath-room and a ward scullery, and on the right a ULANUfca A 1 I rttL nuorl I ALb.
nurses'room. The ward is 45ft. long; 24 ft. wide, and THE ROCHDALE INFIRMAR\ AND DISPENSARY.
12 ft. high. It is intended for 10 cots, so that each cot This institution presents its sixty-eighth annual report,
from which we learn it contained 43 in-
THE ROYAL ISLE of WIGHT INFIRMARY and  patients on January 1st, 1900. The ad-
COUNTY HOSPITAL , RYDE.. . : missions for the year numbered 374,
of which 240 were cured, 62 were re-
lieved, 9 left of their own accord, and 19
died. The daily number of beds occu-
pied was 38, and the average duration of
patients in the hospital was 34 days. The
statistics of the out-patients' department
show that 150 were under treatment at the be-
ginning of the year, and that 2,521 new
patients appeared. Of this total (2,071) 2,404
_ were discharged, 44 died, and 163 remained
-pr??-yf! ''j?Haes_*2a |??.|VVJE ~P under treatment. The number of visits paid
1 " 1 to patients at their homes was 2,919, the con-
sultations were 12,165, and 48 patients were
ground floor plan sent to the convalescent home. The total
income was ?2,944, and the expenditure was
within a few pounds of this sum, but this
satisfactory state of things was more apparent
than real, because one legacy of ?500 from Mr.
Cheetham and another of ?100 from Miss Barns
had to be used as income instead of being in-
vested, which, as the committee remark, is not
altogether satisfactory, and points to the im-
portance of increasing the annual subscriptions.
These amounted to 'only ?505, but the work-
men's committee were able to hand over a sum
of ?610. A table showing the cause of death in
every case is given, and another table shows the
results obtained in the hospital, both in medical
and surgical cases.

				

## Figures and Tables

**Figure f1:**